# Meditation and Compassion Therapy in Psychiatric Disorders: A Pilot Study

**DOI:** 10.7759/cureus.65678

**Published:** 2024-07-29

**Authors:** Cristian I Babos, Giovanni Zucchi, Augusto E Filimberti, Daniel C Leucuta, Dan L Dumitrascu

**Affiliations:** 1 Second Medical Department, Iuliu Hatieganu University of Medicine and Pharmacy, Cluj-Napoca, ROU; 2 Alcoholic Rehabilitation, Ospedale Maria Luigia, Monticelli Terme, ITA; 3 Medical Informatics and Biostatistics Department, Iuliu Hatieganu University of Medicine and Pharmacy, Cluj-Napoca, ROU

**Keywords:** depression, eating disorders, drug addiction, alcohol addiction, compassion, meditation

## Abstract

Introduction

Our study aimed to compare meditation and compassion-based group therapy with the standard of care in patients with eating disorders, drug addiction, alcohol addiction, and depression, concerning acceptance, mindfulness awareness, self-compassion, and psychological distress.

Methods

A controlled designed study was performed, comparing meditation and compassion-focused group therapy added to the standard of care with the standard of care alone, on patients with eating disorders, drug addiction, alcohol addiction, and mood disorders. Four validated questionnaires were administered: the Acceptance and Action Questionnaire-II (AAQ-II), which assesses the ability to be fully in touch with the present moment; the Mindful Attention Awareness Scale (MAAS), which assesses the ability to experience consciously what is happening in the present moment; the Self-Compassion Scale (SCS), which assesses self-compassion characteristics, including loving-kindness; and the Symptom Checklist-90 (SCL-90), which measures psychological distress (anxiety, depression, psychotic behavior, etc.).

Results

There was a total of 75 subjects, out of which 48 represented the experimental group, and 27 represented the control group. The overall mean age of the subjects was 44.8 ± 13.2 years. There were statistically significant increases in the experimental group (baseline vs. end of study) for the AAQ-II, MAAS, and SCS scores, and a statistically significant decrease in the SCL-90 score. In the control group, there was a statistically significant decrease in the SCL-90 score, but no significant differences for other measurements. The comparisons between the two groups at the end of the study were as follows: AAQ-II: 0.7 (-5.74 to 7.15), p = 0.827; MAAS: 4.78 (-3.19 to 12.75), p = 0.233; SCS: 5.89 (-3.18 to 14.96), p = 0.199; SCL-90: -0.26 (-0.62 to 0.1), p = 0.157.

Conclusion

Within the experimental group, all scales improved statistically significantly. There were no statistically significant differences at the end of the study concerning the four scales between the groups. The comparison between groups was limited by data availability.

## Introduction

Eating disorders, drug addiction, alcohol addiction, and depression represent a complex and challenging spectrum of pathologies with an important psychosomatic pattern that collectively impacts millions of individuals worldwide. The aforementioned disorders have important worldwide prevalence: alcohol use disorders (5.1%) [1], drug addiction (4%), with the use of illicit drugs reaching nearly 50% in the Western world (e.g., USA) [2], eating disorders (7.8%) [3], and depressive disorder (5%) [4]. These troubles not only have profound effects on the individuals who experience them, including impaired quality of life, and a heightened risk of comorbid physical and mental health issues, but also present significant societal and healthcare challenges. Effective treatments for these conditions often require a multifaceted approach that addresses not only the symptoms but also the underlying psychological processes contributing to these disorders.

In recent years, there has been growing interest in complementary and alternative approaches to mental health care. There is a particular emphasis on interventions that promote overall psychological well-being, through acceptance, mindfulness awareness, and compassion. Meditation and compassion-based therapies have garnered attention for their potential to enhance emotional regulation, reduce distress, and improve overall mental health outcomes [5].

Meditation is an ancient technique for stilling the mind. It is a relaxed state of attention, either a focused concentration on the mental content (thoughts, feelings, emotions, images, sounds, etc.) or a defocused concentration [6]. One well-known form of meditation, having its roots in the old tradition of Zen Buddhism, is mindfulness. Mindfulness is the awareness that results from purposefully focusing attention on the present moment nonjudgmentally [7]. Awareness is an inherent, fundamental, and distinguishing characteristic of our human nature [7]. The foundation expression of mindfulness is an attitude of observation of witnessing the unfolding mental experience. Mindfulness cultivation is the deliberate practice of directing one’s attention openheartedly and affectionately to experience. This exposes the fundamental inseparability of mindfulness and compassion, as articulated by Kabat-Zinn in 2017 when referring to the Mindfulness-Based Stress Reduction (MBSR) program that he designed [7].

Compassion, which also has its origin in Buddhist philosophy [8], is defined by Goetz et al. [9] as the emotion that comes from seeing another person suffer and inspires a desire to help. Kristin Neff refers to it as an omnidirectional condition that encompasses both oneself and others [10]. Kristin Neff’s theoretical model of self-compassion is comprised of six different elements: increased self-kindness, common humanity, and mindfulness as well as reduced self-judgment, isolation, and overidentification with experience [10].

Mindfulness and self-compassion seem to allow one to face difficult thoughts, feelings, and sensations with the clarity of an open heart [11]. Kuyken et al. (2010) suggested that self-compassion is an essential mediator of outcomes in mindfulness-based interventions [12]. It seems when mindfulness is strengthened, it is naturally compassionate whenever suffering arises [11]. Through mindfulness, the overidentification with experience is lessened by acceptance and by giving up resistance. Letting go of judgementalism, consciously, one realizes that being kind is natural [7].

Meditation practices have shown promise in reducing symptoms of anxiety, depression, and addiction. These practices may enhance emotional regulation and self-awareness [13]. A randomized controlled trial published in JAMA in 2016 by Cherkin et al. on the effect of MBSR vs. cognitive behavioral therapy (CBT) on back pain and functional limitations found improvement in back pain and its functional limitations, with no differences between MBSR and CBT [14]. Also, a meta-analysis and systematic review by Hilton et al. (2017) revealed in their systematic review and meta-analysis that mindfulness meditation has beneficial effects on chronic pain and depression symptoms and quality of life [15]. Garland et al. (2024) found in their randomized controlled trial published in the American Journal of Psychiatry that the Mindfulness-Oriented Recovery Enhancement program decreased chronic pain, opioid use, craving, and opioid cue reactivity in US military personnel undergoing opioid treatment [16]. An umbrella review conducted by Zhang et al. (2024) presented mindfulness-based interventions that significantly improved systolic blood pressure, diastolic blood pressure, smoking, glycosylated hemoglobin, binge eating behavior, depression, and stress [17].

In its turn, self-compassion seems particularly promising in patients suffering from pain and chronic diseases [18-20]. Petrocchi et al. (2023) emphasize that compassion has a significant effect on mental states, affecting social behavior and mood control [21]. Kirby et al. (2017) found in their meta-analysis of randomized controlled trials that compassion-based therapies significantly relieved psychological distress, decreased depression and anxiety, and improved well-being, among patients with a broad spectrum of diagnoses, even when compared to ac­tive control groups [22]. Neff and Germer (2022) wrote in the World Psychiatry journal that self-compassion is demonstrated to be a highly effective instrument to help relieve psychological suffering, changing the lives of patients for the good [23].

It is worth mentioning that Mindful Self-Compassion as the eight-week training program has become one of the most widespread mindfulness-based interventions in the US [24].

The aim of this study was to investigate the effects of meditation and compassion-based group therapy when added to the standard of care, in individuals with eating disorders, drug addiction, alcohol addiction, and depression. We sought to assess the impact of these interventions on key psychological domains, including acceptance, mindfulness awareness, self-compassion, and psychological distress.

## Materials and methods

Study design

A quasi-experimental controlled study was performed. The study compared the effects of meditation and compassion-focused group therapy with standard care in patients with eating disorders (anorexia nervosa and binge disorder), drug addiction, alcohol addiction, and depression.

Participants and setting

The participants were identified from the tertiary hospital "Ospedale Maria Luigia," Monticelli Terme/Parma, Italy. The hospital unit is specialized in mental health and the treatment of addictions and eating disorders. It receives patients from several regions of Italy. Patients were selected from each of the active wards within the hospital: alcohol addiction rehabilitation, toxicological rehabilitation, psychiatric rehabilitation, and eating disorder rehabilitation. Participants aged 18 to 70 years, diagnosed with eating disorders (anorexia nervosa, binge disorder), drug addiction, alcohol addiction, or affective disorders, with minimal aspects regarding the state of consciousness, and the ability to participate in group therapy sessions were eligible for inclusion. Individuals with severe cognitive impairment, acute psychosis, or those unable to provide informed consent were excluded.

Intervention

Experimental Group

Participants in the experimental group received meditation/mindfulness and mindful self-compassion/compassion-focused therapy in addition to standard care. The intervention was offered to groups amounting to 12-15 patients and took place between May and August 2017. The selection of the necessary patients took into account the criterion of covering the entire addictive pathology available at the hospital level, as well as the optimal number considered for a group meeting of about 12-15 patients. Thus, we reached a number of three to four patients for each type of pathology studied: eating disorders, addiction to alcohol and drugs, affective disorders (depression, anxiety), as well as comorbidity or main diagnosis. The four groups of pathologies were chosen to be homogenous between the experimental and control groups. Within each group of pathologies mentioned before, the pathologies were mixed. The intervention consisted of focusing on the awareness of consciousness, manifestation of a compassionate attitude, and acceptance toward one's self. There were mindfulness exercises and compassionate experiential exercises during each session. Most of these consisted of practicing compassion-focused therapy, mindful self-compassion, mindfulness-based therapy, and acceptance and commitment therapy. Each session had a duration of one hour. The first part was dedicated to meditation, centered at its beginning on breathing and body awareness, which took up to 10 minutes. Then the mindfulness part followed, as a guided meditation, enhancing the choice of being aware of everything that comes into experience, letting go of any judgment tendency that appears. The participants were guided progressively through accepting the current of thoughts, feelings, and sensations, without interfering, yet rather observing. This part took another 10 minutes. Then the session continued with compassion-focused exercises comprising writing topics, role games, individual and in pairs, compassionate storytelling, etc., for 30-35 minutes. The session ended with a short closing mindful meditation of usually five minutes. Two experienced psychotherapists, with experience in meditation and compassion-based therapies, conducted the focused groups. The experimental program consisted of daily one-hour therapy sessions, five days a week, for three weeks. Thus, there were four experimental groups during the study, in which the therapeutic program was practically replicated.

Control Group

Participants in the control group received standard care alone. They followed their own therapeutic program as determined by the attending medical team. The intervention consisted of pharmacotherapies and some types of non-pharmacological therapies (e.g. physical exercise, art therapy, and yoga).

Outcomes

The following primary outcomes were assessed using self-administered validated questionnaires at baseline and after the intervention: the Acceptance and Action Questionnaire-II (AAQ-II) scores, the Mindfulness Attention Awareness Scale (MAAS) scores, the Self-Compassion Scale (SCS) scores, and the Symptom Checklist-90 (SCL-90) scores.

The AAQ-II [25] was used to test psychological flexibility. This is the ability to be fully in touch with the present moment, with the thoughts and feelings contained in it, without the need for defensiveness. Depending on the situation, this may involve maintaining or changing behavior in pursuit of values and purpose.

The MAAS [26] was used to evaluate the ability to experience consciously and carefully what is happening in the present moment.

The SCS [27] was used to evaluate self-compassion characteristics: loving-kindness and critical judgment, common humanity and isolation, mindfulness, and over-identification.

The SCL-90 [28] was used to evaluate therapeutic progress in terms of measuring psychological distress (anxiety, depression, psychotic behavior, etc.).

All tests were administered to the participating subjects at the beginning of the intervention and at the end of each three-week group program.

Randomization and allocation

Participants were not randomly assigned but were allocated to groups based on clinical assessments, diagnosis, and treatment availability. No allocation concealment was used.

Blinding

Due to the nature of the interventions, blinding of participants, therapists, and assessors was not feasible.

Statistical analysis

Descriptive statistics, including means and standard deviations, were calculated for continuous variables, while counts and percentages were calculated for qualitative variables. Differences between groups were assessed using unpaired t-tests, while differences between baseline and final observation were assessed using paired t-tests. For all statistical analyses, the 0.05 value was used for statistical significance, as well as two-tailed p-values were computed. No formal sample size calculations were performed. Three experimental groups of about 16 subjects per group were constituted. Statistical analysis was performed using PAST and R environment for statistical computing and graphics (R Foundation for Statistical Computing, Vienna, Austria) version 4.1.2 [29].

Ethical considerations

The study was conducted in accordance with the Declaration of Helsinki and approved by the Ospedale Maria Luigia Management Board, from Monticelli Terme/Parma, Italy, on 29 May 2017. Informed consent was obtained from all participants.

Trial registration and reporting

The study adheres to the CONSORT (Consolidated Standards of Reporting Trials) guidelines for reporting clinical trials. No public trial registration was pursued. The study protocol was approved by the Ospedale Maria Luigia Management Board.

## Results

There was a total of 75 subjects, out of which 48 represented the experimental group, and 27 represented the control group. The overall mean age of the subjects was 44.8 ± 13.2 years. The age difference between the experimental and control groups was statistically significant, with the former being 6.4 years older (Table 1). There were no significant differences concerning sex; the proportion of women in the experimental group was slightly higher than those in the control group. Concerning the diagnosis, there were no significant differences, with both groups having similar eating disorders and drug and alcohol addictions, as well as mood disorders.

**Table 1:** Patients’ characteristics

Characteristic	Experimental group (n = 48)	Control group (n = 27)	P-value
Age (years), mean (SD)	42.5 (13.2)	48.9 (12.2)	0.040
Female, n (%)	30 (62.5)	14 (51.9)	0.369
Male, n (%)	18 (37.5)	13 (48.1)	
Diagnosis, n (%)			0.930
Eating disorders	13 (27.1)	6 (22.2)	
Drug addiction	5 (10.4)	4 (14.8)	
Alcohol addiction	11 (22.9)	6 (22.2)	
Mood disorders	19 (39.6)	11 (40.7)	

The baseline characteristics of the participants in both groups were similar regarding the MAAS and SCL-90 scores (Table 2). The SCS scores were significantly higher in the control group compared to the experimental group. The AAQ-II scores were higher in the control group compared to the experimental group, albeit not reaching the significance level.

**Table 2:** Baseline psychometric characteristics AAQ-II, Acceptance and Action Questionnaire-II; MAAS, Mindfulness Attention Awareness Scale; SCS, Self-Compassion Scale; SCL-90, Symptom Checklist-90.

Characteristic	Experimental group (n = 48)	Control group (n = 27)	P-value
AAQ-II, mean (SD)	30.17 (8.83)	35.89 (14.39)	0.069
MAAS, mean (SD)	56.17 (15.11)	58.81 (19.80)	0.518
SCS, mean (SD)	58.29 (15.69)	68.33 (18.11)	0.014
SCL-90, mean (SD)	1.61 (0.78)	1.46 (0.89)	0.449

The experimental group exhibited statistically significant increases in AAQ-II, MAAS, and SCS scores from baseline through the end of the trial (Table 3). Additionally, there was a statistically significant drop in the SCL-90 scores. The control group exhibited a statistically significant reduction in the SCL-90 scores, whereas no significant differences were seen for the remaining data.

**Table 3:** Differences within groups between the baseline and final measurements AAQ-II, Acceptance and Action Questionnaire-II; MAAS, Mindfulness Attention Awareness Scale; SCS, Self-Compassion Scale; SCL-90, Symptom Checklist-90.

Characteristic	Experimental group (n = 48)	Control group (n = 27)
AAQ-II, mean (SD)	<0.001	0.124
MAAS, mean (SD)	<0.001	0.63
SCS, mean (SD)	<0.001	0.296
SCL-90, mean (SD)	<0.001	0.016

Within the experimental group the AAQ-II, MAAS, and SCS scores significantly increased. The same scores increased in the control group as well but were not statistically significant. The score increases were evidently higher in the experimental group compared to the control group. The SCL-90 scores significantly decreased in both groups. The decrease in the SCL-90 scores was more important in the experimental group compared to the control group.

All observed final mean scores in the experimental group, concerning AAQ-II, MAAS, and SCS, were higher compared to those in the control group, yet they did not reach the significance level (Table 4). Similarly, the mean SCL-90 score was lower in the experimental group compared to the control group, still without being statistically significant.

**Table 4:** End of study differences between the groups AAQ-II, Acceptance and Action Questionnaire-II; MAAS, Mindfulness Attention Awareness Scale; SCS, Self-Compassion Scale; SCL-90, Symptom Checklist-90.

Characteristic	Experimental group (n = 48)	Control group (n = 27)	Mean difference (95% CI)	p-value
AAQ-II, mean (SD)	39.33 (8.59)	38.63 (15.21)	0.7 (-5.74 to 7.15)	0.827
MAAS, mean (SD)	64.67 (11.96)	59.89 (18.41)	4.78 (-3.19 to 12.75)	0.232
SCS, mean (SD)	76.37 (17.4)	70.48 (21.41)	5.89 (-3.18 to 14.96)	0.199
SCL-90, mean (SD)	0.91 (0.67)	1.17 (0.89)	-0.26 (-0.62 to 0.1)	0.157

**Figure 1:**
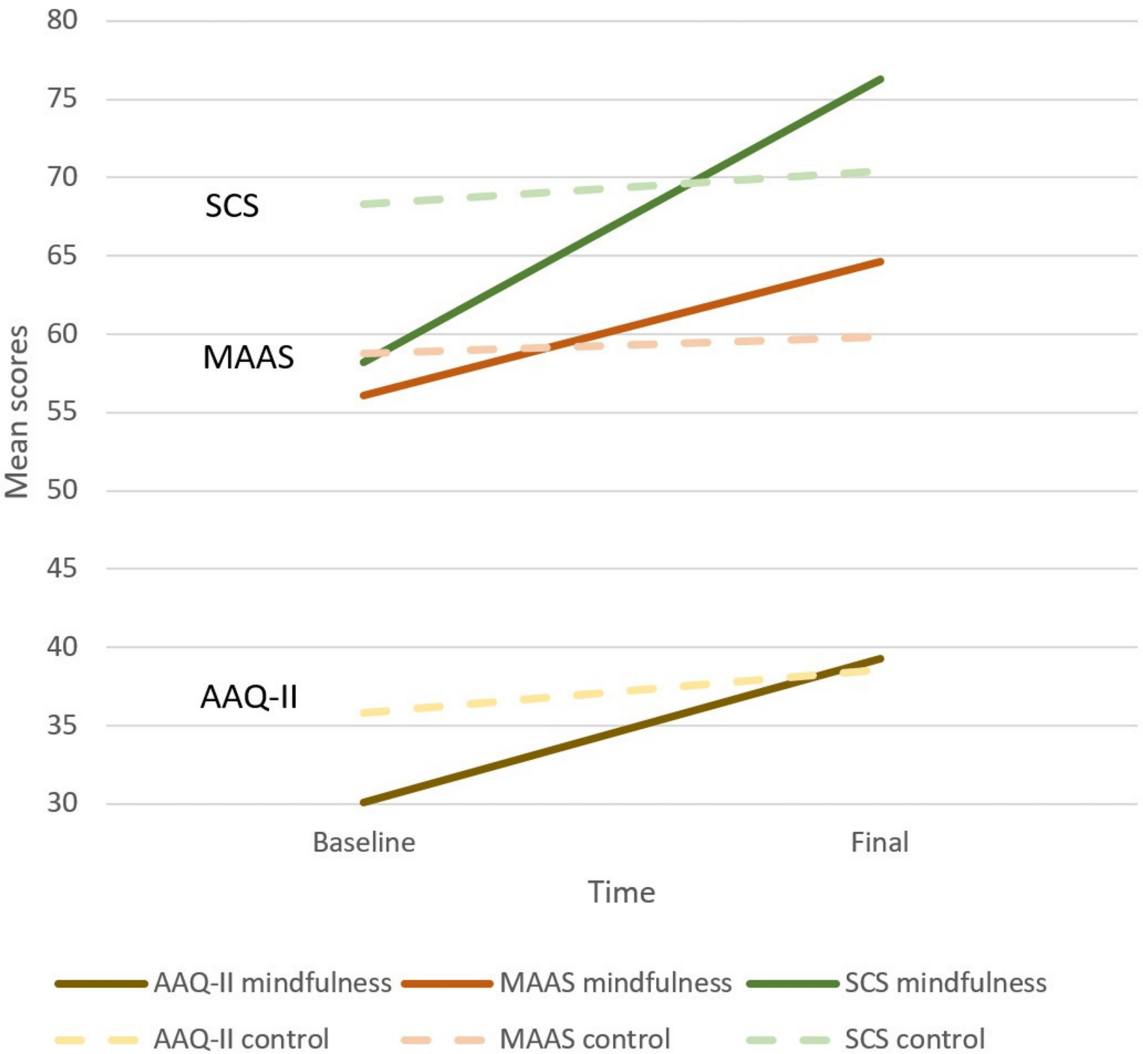
AAQ-II, MAAS, and SCS mean scores evolution for mindfulness and control group AAQ-II, Acceptance and Action Questionnaire-II; MAAS, Mindfulness Attention Awareness Scale; SCS, Self-Compassion Scale.

**Figure 2:**
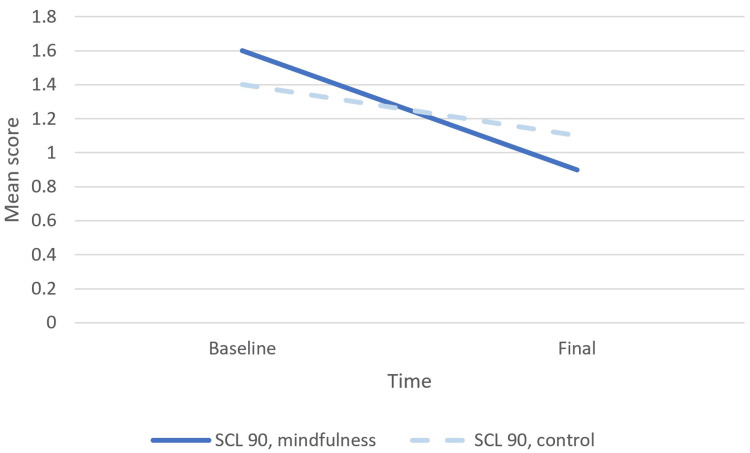
SCL-90 mean scores evolution for mindfulness and control group SCL-90, Symptom Checklist-90.

The evolution of the scores of interest in time is presented in Figures 1, 2.

## Discussion

The aim of this study was to investigate the effects of meditation and compassion-based group therapy when added to the standard of care in patients with eating disorders, drug addiction, alcohol addiction, and mood disorders. We assessed the impact of these interventions on acceptance, mindfulness awareness, self-compassion, and psychological distress in comparison to a control group receiving only standard care. The baseline psychological-mental status from the point of view of awareness and compassion, as measured by the AAQ-II, MAAS, and SCS, was better in the control group compared to the experimental group. The experimental group improved significantly on these lines, compared to the control group, which had minor improvements that were not statistically significant. At the end of the study, all these measures were better in the experimental group compared to the control group but did not reach the significance level. The experimental group demonstrated improvement that ultimately surpassed the previously "healthier" control group, as depicted in Figure 1. The baseline psychological distress, as measured by SCL-90, was higher in the experimental group compared to the control group. In both groups, the psychological distress in time diminished considerably in the experimental group. At the end of the study, this indicator was lower in the experimental group compared to the control group, but the difference was not statistically significant. In the abstract, the improvements in the experimental group were more important compared to the control group, but since at baseline, the status of the experimental group was poorer compared to the control group; the final measurements were not significantly different. Due to the absence of data regarding the direct comparison of changes in the outcomes of interest between the two groups, the superiority of the experimental intervention could only be suggested.

The mean baseline age in the control group was higher than that in the experimental group. A growing body of research highlights a positive correlation between advancing age and heightened levels of acceptance. Shallcross et al. (2013) observed a positive association between increased age and acceptance in a community sample with age spanning six decades [30]. Schirda et al. (2016) found that in a cohort of older adults, acceptance was more frequently used than in a cohort of younger adults [31]. This could explain why higher scores were found at baseline in the control group compared to the experimental one.

AAQ-II

In the experimental group, there was a statistically significant increase in the AAQ-II scores from baseline to the end of the study. This suggests that individuals participating in meditation and compassion-focused group therapy showed greater improvements in their levels of acceptance compared to those in the control group. In a study by Mathur et al., the AAQ-II score increased by 40.8% in a series of patients with late-life depression following mindfulness-based cognitive therapy [32], observations that are in line with our results. Enhancing mindfulness abilities and fostering acceptance as measured by the AAQ-II can facilitate healing in patients with treatment-resistant depression [33]. In a study by Marchand et al. [34], veterans experiencing mental health issues and/or substance use problems participated in a program that combined outdoor activities like sailing with mindfulness exercises. Comparisons within the same individuals showed notable improvements in the AAQ-II scores from before to after the program, a finding that is concordant with our results. While the statistical analysis using Poisson regression did not show significant results, there was a decrease in the number of visits to substance abuse treatment centers following the program. A study by Kaplan et al. [35] examined how effective mindfulness-based resilience training was in reducing burnout and alcohol consumption among law enforcement officers. Before and after comparisons showed a significant reduction in the AAQ-II scores within the group that received the intervention, indicating improved psychological flexibility similar to our findings. Although there was an increase in the SCS short-form scores, which measure self-compassion, this change was not statistically significant. The study found that increased mindfulness was a significant predictor of reduced problematic alcohol use after the intervention.

MAAS

Similar to the AAQ-II, the experimental group showed a statistically significant increase in the MAAS scores, indicating end-of-intervention improved mindfulness awareness compared to baseline. This finding suggests meditation and compassion-focused therapy may enhance mindfulness awareness, but this effect was not significantly different from the improvements seen in the control group. Mindfulness as measured by MAAS (but not AAQ-II) was found to be inversely associated with drug addiction in the case of current opioid misuse in a chronic back pain sample, as found by Villarreal et al. [36]. Research conducted by Baptista et al. [37] discovered a link between reduced mindfulness and the likelihood of an individual being a medium to high-risk consumer of sedative-hypnotic medication. In a pilot randomized study by Beattie et al. [38] on a sample of participants attending a maternity service, the MAAS score improved in both mindfulness-integrated cognitive behavioral therapy and control group at the end of the interventions, yet six weeks later, the MASS score still continued to increase in the mindfulness group, but not in the control group. Nevertheless, the depression scores were not statistically significantly different. A progressive self-focus meditation intervention in a study by Leite et al. [39] significantly decreased anxiety and depression scores, and increased MAAS scores compared to the waiting list control group.

SCS

The experimental group demonstrated a statistically significant increase in the SCS scores from baseline to the end of the study, indicating enhanced self-compassion. Self-compassion, as measured by the SCS, has been identified by Van Dam et al. [40] as a robust predictor of symptom severity and quality of life in anxiety, accounting for significant variance in outcomes. Lopez et al. [41] found that the negative items of SCS significantly predicted depressive symptoms and negative affect. The same study found that mindfulness measured by another instrument, the Five Facet Mindfulness Questionnaire (FFMQ), significantly predicted negative affect, positive affect, and depressive symptoms. FFMQ measures mindfulness in more detail compared to MAAS, being more nuanced in its appraisal. In a randomized controlled trial by Jazaieri et al. [42] on participants with generalized social anxiety disorder, a mindfulness-based stress reduction intervention significantly reduced social anxiety symptom severity and depression and increased SCS scores compared to the untreated group or aerobic exercise group. In a systematic review with meta-analysis on meditation and irritable bowel syndrome by Babos et al. [43], the spiritual scale scores significantly improved in the meditation group compared to the control one.

SCL-90

Both the experimental group and the control group showed a statistically significant decrease in SCL-90 scores, indicating a reduction in psychological distress. The experimental group had higher changes in SCL-90 scores. However, there was no statistically significant difference between the two groups in SCL-90 scores at the end of the study. A study by Luo et al. [44] found a statistically significant decrease in the SCL-90 scores in the experimental group that followed Huatou Chan training (a Chinese Zen meditation practice), compared to the control group in a healthy subject’s cohort. Furthermore, a randomized controlled trial by Sundquist et al. [45] found mindfulness-based group therapy had a higher reduction in SCL-90 scores compared to the treatment as usual control group, in a population with a broad range of psychiatric symptoms, including depression, anxiety, and adjustment disorders. Another randomized controlled trial by Lappalainen et al. [46] on a sample of subjects with depression symptoms found a significant reduction in SCL-90 scores in web-based acceptance and commitment therapy (that implies meditation approaches), adjuvant to pharmacotherapy, compared to waiting list control group. Finally, a randomized study by Lee et al. [47] on patients with anxiety disorder found that those patients who received a meditation-based stress management program had significantly lower scores on the SCL-90 anxiety and hostility subscale compared to the patients receiving an anxiety disorder education program, although no difference was observed concerning the SCL-90 phobic anxiety subscale.

Limitations

Our study offers insights into meditation and therapy for various disorders, but its limitations should be considered in interpreting the findings. The study's small sample size may not capture the diversity of individuals with complex mental health conditions. The quasi-experimental design can introduce a potential selection bias, affecting the quality of outcomes. Some data were not available for analysis regarding the changes before and after the intervention. This might hinder a possible statistically significant difference that could not be shown by comparing only the post-treatment endpoints. Furthermore, confounding using this design cannot be controlled, as in randomized controlled trials. Heterogeneity among participant disorders could lead to variable treatment responses. Variability in standard care for the patients might have influenced the results. Reliance on self-report measures could introduce response bias and may not fully capture participants' experiences. The blinding is not feasible for such type of study; thus subjectivity of the patients can influence the reporting (e.g., placebo effect). The study's specific clinical setting may limit the generalizability of the findings to other contexts. The brevity of the study timeframe (three weeks) might have hindered the potentially more substantial beneficial effects of the mindfulness intervention and could explain why the study did not reach the significance threshold. Nevertheless, we tried to compensate for the short timeframe with daily one-hour interventions. This approach was different from other studies on the topic of applied meditation therapy, which uses about two hours of intervention per week, but in longer timeframes [43]. The short-term follow-up period limits understanding of the interventions' long-term effects.

Concerning the fact our study did not use a long-term follow-up evaluation after the end of the study intervention, a short review of the literature on longer-term effects seems appropriate. In a meta-analysis by Goldberg et al. (2019), mindfulness outperformed no treatment comparisons for depression, pain, and schizophrenia and was equivalent or superior to other active treatments for addictions, depression, pain, and weight/eating. The average follow-up length post-treatment was 6.43 months for the studies where it was available [48]. In a large and very methodologically rigorous randomized controlled trial of mindfulness-based relapse prevention (MBRP) published by Bowen et al. (2014) [49], at the 12-month follow-up, MBRP participants reported significantly fewer days of substance use and significantly decreased heavy drinking compared with cognitive behavioral relapse prevention and usual care. In a randomized controlled study by Witkiewitz et al. (2019), it is shown that drinking reductions associated with improvements in functioning and also self-reported craving improved statistically significantly in the MBRP group compared with an active treatment group, both at the end of the study and at two-month follow-up [50]. In a pilot study by Duarte et al. (2017) on mindfulness and self-compassion in patients with binge-eating disorder, the results were significant at the end of the study and one-month follow-up, regarding the improvement among others of self-reported binge-eating symptomatology, general eating psychopathology, overvaluation of body weight and shape, and symptoms of depression and stress [51].

Strengths

Despite the limitations mentioned, our study offers several noteworthy strengths that contribute to its scientific and clinical significance. The study explores non-pharmacological interventions that have the potential to complement standard care and improve the overall well-being of these patients. Employing a comprehensive, multi-dimensional assessment approach using four well-established validated psychological scores enhances understanding of treatment effects. Comparing interventions with standard care provides insights into their incremental benefits. Investigating psychosocial factors like acceptance and mindfulness, which have gained increasing attention in the mental health field, contributes to mental health resilience, quality of life, and well-being. The study's focus on enhancing acceptance, mindfulness, and self-compassion aligns with a patient-centered approach that empowers individuals to take an active role in their health care.

While no statistically significant differences were observed between the experimental and control groups at the end of the study, the significant improvements within the experimental group suggest the potential clinical benefits of meditation and compassion-focused group therapy.

This study contributes as a groundwork for future research in the field of meditation, compassion-focused therapy, and mental health treatment. The results provide a basis for more extensive, controlled trials with larger and more diverse populations, as well as longer follow-up periods to assess the sustainability of improvements.

## Conclusions

In summary, the results of this quasi-experimental pilot study indicate that within the experimental group, participants experienced statistically significant improvements in acceptance, mindfulness awareness, and self-compassion following meditation and compassion-focused group therapy, as well as a significant reduction in psychological distress. However, when compared to the control group, receiving standard care alone, no statistically significant differences were found in the outcomes of these four scales. The comparison between groups was limited by the data availability.
